# Prevalence of COPD and Tobacco Smoking in Tunisia — Results from the BOLD Study

**DOI:** 10.3390/ijerph10127257

**Published:** 2013-12-17

**Authors:** Hager Daldoul, Meriam Denguezli, Anamika Jithoo, Louisa Gnatiuc, Sonia Buist, Peter Burney, Zouhair Tabka, Imed Harrabi

**Affiliations:** 1 Laboratory of Physiology, Faculty of Medicine Ibn El Jazzar, Mohamed Karoui Avenue, Sousse 4000, Tunisia; E-Mails: hagerdaldoul@yahoo.fr (H.D.); tabkazouhair@yahoo.fr (Z.T.); 2 National Heart and Lung Institute, Imperial College London, Emmanuel Kaye Building, 1 Manresa Road, London, SW3 6LR, UK; E-Mails: a.jithoo@imperial.ac.uk (A.J.); l.gnatiuc2@imperial.ac.uk (L.G.); p.burney@imperial.ac.uk (P.B.); 3 Department of Pulmonary and Critical Care Medicine, Oregon Health and Science University, Portland, OR 97239, USA; E-Mail: buists@ohsu.edu; 4 Department of Epidemiology, University Hospital Farhat Hached, Sousse 4000, Tunisia; E-Mail: imed_harrabi@yahoo.fr

**Keywords:** COPD, prevalence, smoking, Tunisia, BOLD

## Abstract

In Tunisia, there is a paucity of population-based data on Chronic Obstructive Pulmonary Disease (COPD) prevalence. To address this problem, we estimated the prevalence of COPD following the Burden of Lung Disease Initiative. We surveyed 807 adults aged 40+ years and have collected information on respiratory history and symptoms, risk factors for COPD and quality of life. Post-bronchodilator spirometry was performed and COPD and its stages were defined according to the Global Initiative for Chronic Obstructive Lung Disease (GOLD) guidelines. Six hundred and sixty one (661) subjects were included in the final analysis. The prevalence of GOLD Stage I and II or higher COPD were 7.8% and 4.2%, respectively (Lower Limit of Normal modified stage I and II or higher COPD prevalence were 5.3% and 3.8%, respectively). COPD was more common in subjects aged 70+ years and in those with a BMI < 20 kg/m^2^. Prevalence of stage I+ COPD was 2.3% in <10 pack years smoked and 16.1% in 20+ pack years smoked. Only 3.5% of participants reported doctor-diagnosed COPD. In this Tunisian population, the prevalence of COPD is higher than reported before and higher than self-reported doctor-diagnosed COPD. In subjects with COPD, age is a much more powerful predictor of lung function than smoking.

## 1. Introduction

Chronic obstructive pulmonary disease (COPD) represents a major public health problem in developing countries and especially in North Africa [1]. It is characterized by lung function impairment with airway obstruction, and is currently estimated to be one of the leading causes of death in 2010 [2]. Although COPD is one of the leading causes of mortality and morbidity, epidemiological data on COPD are very limited in North Africa, including Tunisia. The comparison of the few Tunisian COPD prevalence estimates with the international literature showed that estimated prevalence of COPD in Tunisia was low compared with America and Europe and the disease is certainly under diagnosed [1]. In fact, National estimates of COPD prevalence are usually based on self-reported diagnosis without the use of objective measurement of lung function by spirometry testing. One survey of chronic bronchitis has estimated the prevalence as 3.8% (1.1% in women and 6.6% in men) [3]. 

Several investigations, using spirometry, and conducted in the United States [4], Korea [5], Spain [6], Sweden [7], and South America [8], have demonstrated the under-diagnosis of COPD. The most extreme example was observed in Japan, where the results of the 2004 population-based prevalence of COPD survey contrasts with the estimates of the Japanese Ministry of Health (10.9% *vs.* 0.3% respectively) [9]. Only 9.4% of the subjects documented with airflow obstruction reported a physician diagnosis of COPD. Similar rates of under diagnosis have been frequently reported [10].

Therefore, objective measurement of lung function by spirometry testing is needed to determine the true prevalence of COPD in Tunisia. The Burden of Obstructive Lung Disease (BOLD) study was designed to provide a standardized framework for estimating COPD prevalence, risk factors and economic burden in different countries around the world [11]. In this paper we report the population estimate of COPD prevalence in Sousse, Tunisia, using the BOLD protocol. 

## 2. Experimental Section

We followed the BOLD protocol as it has been described elsewhere [11,12]. Data were collected by trained and certified staff, under continuous quality control from the BOLD coordinating center.

### 2.1. Participants

The survey was conducted on a gender-stratified representative random sample of non-institutionalized residents selected from the general population living in the urban area of Sousse. Two quartiers with clear administrative boundaries were selected for convenience and districts were sampled at random from each of the two selected quartiers. Site or home visits were scheduled for adults aged ≥40 years to complete questionnaires and perform pre- and post-bronchodilator spirometry. All participants gave written informed consent, and the study was approved by the Medical School of Sousse Ethics’ Committee.

### 2.2. Data Collection

#### 2.2.1. Study Outcomes

Spirometry was performed according to ATS (American Thoracic Society) criteria [13,14] before and 15–60 min after administering 200 µg of salbutamol (Ventolin, GlaxoSmithKline, Middlesex, UK). Portable spirometers (Easy One ndd. Medizintechnik, Zurich, Switzerland) were used in this study and were daily calibrated, using a 3.00 L syringe. All spirometry data were reviewed and graded for quality by the BOLD Pulmonary Function Quality Control Centre. We defined COPD according to GOLD (Global Initiative for Chronic Obstructive Lung Disease) criteria, as post-bronchodilator FEV_1_/FVC (FEV_1_: Forced expiratory volume in 1 s; FVC: Forced vital capacity) less than 70% [15].

#### 2.2.2. Definition of COPD Stages

COPD stages in those with post-bronchodilator (post-BD) FEV_1_/FVC <0.7, were defined according to GOLD guidelines: Stage I: if FEV_1_ ≥80% predicted; Stage II: if FEV_1_ ≥50 and <80% predicted; Stage III: if FEV_1_ ≥30 and <50%; and Stage IV: if FEV_1_<30% predicted. We used the third US National Health and Nutrition Examination Survey (NHANES 3) to compute predicted values for FEV_1_ [16]. We examined also the impact of using FEV_1_/FEV_6_ (FEV_6_: Forced expiratory volume in 6 s) in place of FEV_1_/FVC in our definitions [17]. Doctor-diagnosed COPD was defined as self-reported physician’s diagnosis of COPD, chronic bronchitis, or emphysema. 

The number of pack-years of cigarette smoking was calculated as the average number of cigarettes smoked per day divided by 20, times the duration of smoking in years.

Education level was assessed as self-reported years of education and classified according to the education system in Tunisia, as 0, 1–5, 6–8, 9–11 and >12 years.

#### 2.2.3. Questionnaire Data

The questionnaires used in this study contained information on history of respiratory symptoms and diseases, use of respiratory medication, comorbidities, risk factors for COPD, health-care utilization, tobacco exposure, use of biomass fuels for cooking or heating, occupational exposures and activity limitation due to breathing problems [11].

### 2.3. Statistical Analysis

Population estimated prevalence of COPD was calculated for the overall Sousse population, using population weights. Prevalence of COPD was stratified by gender, age and pack-years of cigarette smoking. 

The significance of differences between proportions was determined by chi-square tests. Calculations of odd ratios (ORs) and 95% CI values for COPD in relation to potential risk factors were performed with multivariate logistic regression models. The variables of sex, age groups, body mass index (BMI), smoking status, pack-years of smoking, occupational exposure to dusts/gases/fumes, respiratory disease in family, pulmonary problems in childhood and education were tested in the multivariate logistic regression model. All statistical tests were performed with Stata statistical software (version 7.0; Stata Corporation, College Station, TX, USA), and a *p* value of 0.05 was considered significant. 

## 3. Results

### 3.1. Sample Demographics

Of the 807 subjects sampled from Sousse region in Tunisia, 717 were interviewed. The response rate was 90%. The number of non-responders and ineligible participants were 77 and 13, respectively. The reasons for non-response included refusals, contact failures, spirometry ineligibility, and failed attempts.

**Figure 1:**
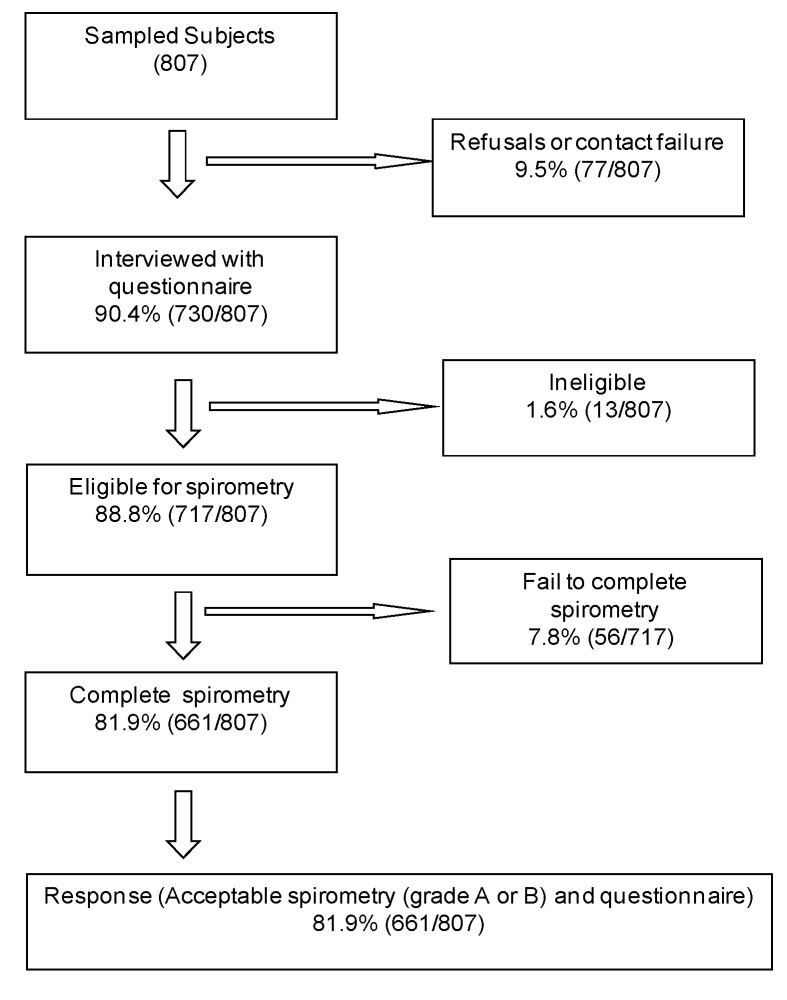
Response rate of questionnaire and spirometry.

Among the 717 interviewees, 56 failed to complete the spirometry testing and 661 completed acceptable and reproducible post-BD spirometry and questionnaires and were included in this analysis (Figure 1). There were no significant differences in age, sex and smoking status between responders and non-responders, thus, the pattern of these variables distribution was similar in the two groups, suggesting that the study participants are highly representative of the general population (data not shown). 

The study sample consisted of 309 men and 352 women. The mean age of the final study population did not differ significantly between men and women. The educational level differs significantly between men and women (2.8 (SD, 1.23) *vs.* 1.9 (SD, 1.41); *p* < 0.01). 

A history of current or past smoking was greater in men than women (47.4% *vs.* 7.0% and 74.3% *vs.* 8.5%, respectively). Despite the differences in smoking history between men and women, percent of predicted FEV_1_ and FVC did not differ between sexes while FEV_1_/FVC was significantly higher in women (Table 1).

Moreover, we used spirometric data to classify people with restricted spirometry (FEV1/FVC ≥ 0.70 and FEV1 < 80%). Overall, we found that 12.6% of men (39/309) and 19% of women (67/352) had restricted spirometry.

**Table 1:** Participants smoking status * and lung function ** by sex.

	Men	Women	*p*-value
Smoking status (pack years)			<0.001
Never	78 (19.6)	320 (80.4)	
0–10	22 (62.9)	13 (37.1)	
10–20	57 (87.7)	8 (12.3)	
20+	152 (93.3)	11 (6.7)	
Lung Function			
FEV_1_ (L)	3.1 (0.77)	2.3 (0.60)	<0.001
FEV_1_ (%, predicted)	90.5 (17.34)	92.3 (17.10)	0.1913
FVC (L)	4.0 (0.79)	2.8 (0.66)	<0.001
FVC (%, predicted)	89.3 (13.66)	88.7 (14.63)	0.5801
FEV_1_/FVC (%)	77.8 (8.66)	81.9 (5.73)	<0.001

* Smoking pack-years are expressed in N (%); ** Lung function measures are taken post bronchodilator and are expressed in Mean (SD); Abbreviations: FEV_1_—1 s forced expiratory volume, FVC—forced vital capacity, SD—standard deviation.

Table 2 shows the estimated prevalence of smoking in Tunisia by age and gender. 41.9% of the Tunisian population is estimated to be ever smokers, while the prevalence of current smoking in this population is estimated to be 28.6%. Current smokers accounted for 49.4% of the male subjects and 7.3% of the female subjects.

According to GOLD diagnostic criteria, the overall prevalence of stage I or higher COPD was 7.8% (1.2) (Lower Limit of Normal (LLN) modified stage I or higher COPD prevalence was 5.3% (1.4)).

**Figure 2:**
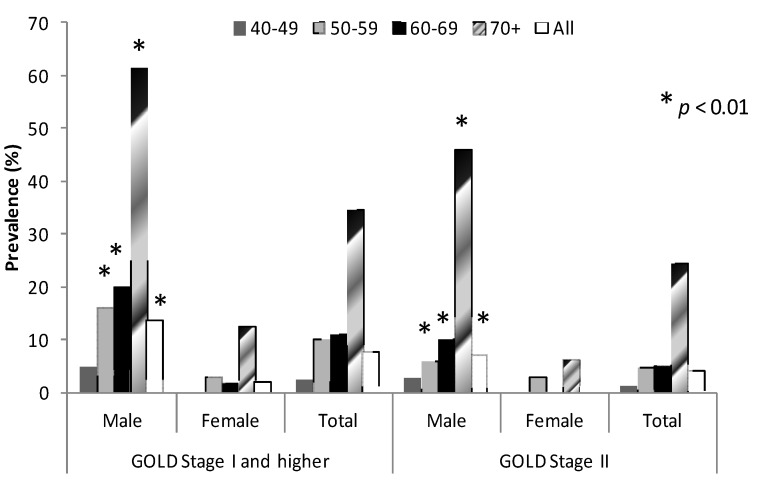
Prevalence of COPD (GOLD stage I and II COPD) by gender and age groups.

The prevalence of COPD was significantly higher in men than in women (13.5% (2.9) *vs.* 1.9% (0.7) respectively; *p* < 0.01). The prevalence of GOLD stage II COPD was 4.2% (0.9) (LLN modified stage II COPD prevalence was 3.8% (1.3)) and was also different between men and women (7% (1.9) *vs.* 1.2% (0.7) respectively; *p* < 0.01) (Figure 2). However, none of the study subjects met criteria for GOLD stages III or IV COPD. The prevalence of COPD stage I and stage II increased with age in both sexes, and for each age group was greater in men than in women (*p* < 0.01) (Figure 2).

**Table 2:** Population estimates of the smoking distribution in Sousse, Tunisia, by age and gender *.

	40–49 year	50–59 year	60–69 year	70+ year	All
Male gender					
Never smoker	24.3 (6.7)	23.5 (3.5)	34.4 (5.4)	25.0 (7.2)	25.8 (4.1)
Former smoker	16.0 (3.6)	26.9 (4.0)	42.0 (6.2)	37.6 (7.7)	24.8 (3.0)
Current smoker	59.6 (5.8)	49.6 (4.8)	23.6 (3.8)	37.5 (7.8)	49.4 (4.2)
Female gender					
Never smoker	89.4 (3.2)	90.7 (3.0)	96.2 (2.8)	95.2 (4.7)	91.3 (2.1)
Former smoker	1.5 (1.0)	2.8 (1.6)	0	0	1.5 (0.7)
Current smoker	9.2 (2.8)	6.5 (2.1)	3.8 (7.8)	4.8 (4.7)	7.3 (1.9)
Total					
Never smoker	57.1 (4.6)	54.9 (3.1)	64.6 (3.5)	64.2 (5.6)	58.1 (2.8)
Former smoker	8.7 (2.0)	15.6 (2.1)	21.5 (3.5)	16.6 (3.7)	13.3 (1.4)
Current smoker	34.2 (4.1)	29.5 (3.2)	13.9 (2.5)	19.2 (4.8)	28.6 (2.9)

* All values are % (SE); Abbreviations: SE—standard error.

COPD: Chronic Obstructive Pulmonary Disease; GOLD I: Global Initiative for Chronic Obstructive Lung Disease Stage I COPD; GOLD II: Global Initiative for Chronic Obstructive Lung Disease Stage II COPD; * *p* < 0.01, significant difference between men and women according to age.

**Figure 3:**
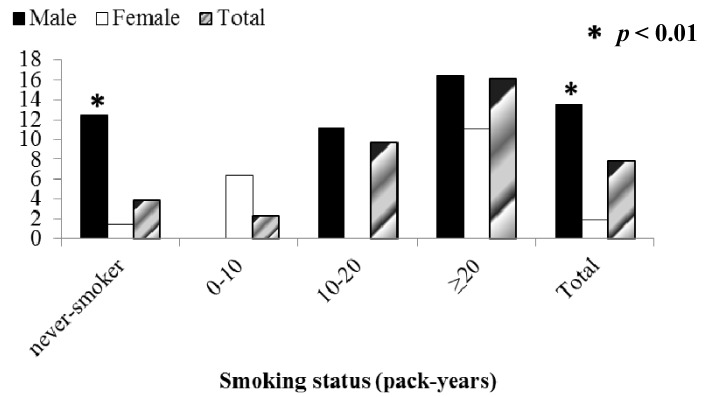
Prevalence of GOLD Stage I and higher by pack years and sex.

**Figure 4:**
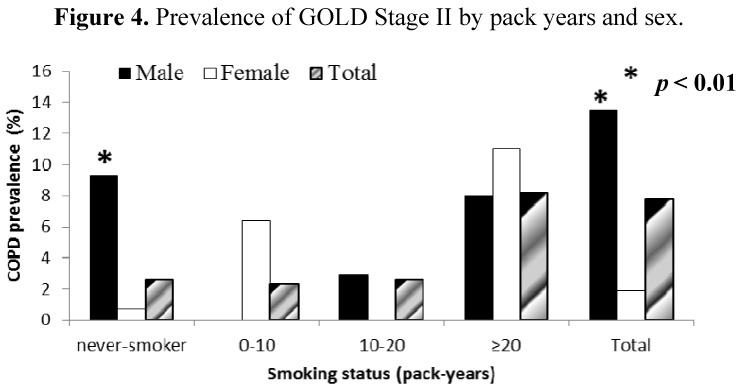
Prevalence of GOLD Stage II by pack years and sex.

Our study showed that 74.5% of patients with COPD (94.7% of male and 5.2% of female patients with COPD) were smokers. As expected, prevalence of COPD (GOLD stage I and higher) increased with increasing pack-years of cigarette smoking in both men and women from 3.9% in subjects who had never smoked to 16.1% in those with a smoking history ≥20 pack-years. Similarly, the prevalence of GOLD stage II COPD increased from 2.6% in subjects who had never smoked to 8.2% in those with the most pack-years of smoking (Figure 3 and Figure 4). Surprisingly, high prevalence of COPD stages I and II was found in never-smokers and especially in men (12.4 % (6.7) and 9.3% (5.4), for stages I and II respectively; *p* < 0.01) (Figure 3 and Figure 4).

GOLD I: Global Initiative for Chronic Obstructive Lung Disease Stage I COPD; * *p* < 0.01, significant difference between men and women according to smoking status.

GOLD II: Global Initiative for Chronic Obstructive Lung Disease Stage II COPD; * *p* < 0.01, significant difference between men and women according to smoking status.

### 3.2. Risk Factors for COPD

We performed univariate and multivariate logistic regression to assess the association of COPD with gender, age, education, smoking history, BMI, childhood and family history of respiratory disease and occupational exposure to dust. After mutual adjustment for all these potential factors in the model, we found that in our study population, COPD was more common in subjects aged 70+ years (OR = 17.67, *p* = 0.007) compared to subjects aged 40–49 years of age, and in those with a BMI < 20 kg/m^2^ (OR = 6.61, *p* = 0.02) compared to subjects with a BMI of 20–25 kg/m^2^. Smoking 10+ pack years per year, was independently associated with an increased risk of COPD (OR = 1.25, *p* = 0.003), however that association decreased and did not reach conventional levels of statistical significance (OR = 1.18, *p* = 0.1) after adjustment of all other potential risk factors in the model (Table 3).

**Table 3:** Factors associated with COPD *.

	Unadjusted OR (95% CI)	*p*-value	Adjusted OR (95% CI) **	*p*-value
Sex				
Male	1		1	
Female	0.198 (0.062–0.635)	0.010	0.201 (0.015, 2.733)	0.210
Age, years				
40–49	1		1	
50–59	2.090 (0.769–5.677)	0.137	2.105 (0.755, 5.863)	0.142
60–69	2.472 (0.778–7.853)	0.116	3.519 (0.942, 13.152)	0.060
≥70	10.403 (2.072–52.222)	0.007	17.670 (2.488, 125.472)	0.007
Education, years				
0	1		1	
1–5	0.375 (0.076–1.840)	0.208	0.480 (0.062, 3.698)	0.455
6–8	0.774 (0.211–2.844)	0.681	1.471 (0.245, 8.827)	0.653
9–11	0.798 (0.329–1.934)	0.595	0.932 (0.112, 7.761)	0.944
≥12	0.602 (0.142–2.545)	0.464	0.881 (0.273, 2.839)	0.820
Smoking Status				
Never smoker	1			
Former smoker	2.164 (0.667, 7.022)	0.182	0.426 (0.041, 4.429)	0.449
Current smoker	3.301 (1.127, 8.150)	0.030	0.641 (0.084, 4.885)	0.648
Smoking pack-years				
10 year increase	1.252 (1.093–1.435)	0.003	1.176 (0.941, 1.470)	0.141
Body Mass Index				
<20	4.673 (1.032, 21.159)	0.046	6.610 (1.439, 30.354)	0.019
20–25	1		1	
25–30	0.536 (0.145, 1.977)	0.324	0.821 (0.267, 2.527)	0.714
30–35	0.743 (0.282, 1.960)	0.524	1.299 (0.401, 4.208)	0.642
>35	0.271 (0.069, 1.063)	0.060	0.660 (0.177, 2.466)	0.512
Occupational dust exposure				
10 year increase	1.288 (0.978, 1.697)	0.069	0.996 (0.689, 1.441)	0.983
Childhood breathing problems				
No	1		1	
Yes	0.956 (0.099, 9.242)	0.967	1.666 (0.195, 14.235)	0.620
Family history of pulmonary disease				
No	1		1	
Yes	0.190 (0.024, 1.508)	0.108	0.194 (0.018, 2.114)	0.164

* Post-bronchodilator FEV_1_/FVC < Lower Limit of Normal (LLN) defined COPD; ** Mutual adjustment for all the risk factors in the table; Abbreviations: CI = confidence interval; COPD = chronic obstructive pulmonary disease; OR = odds ratio.

### 3.3. Lifetime Diagnosis of COPD and Respiratory Symptoms

The prevalence of self-reported doctor-diagnosed chronic bronchitis, emphysema or COPD was 3.5% (0.7). This value is half of the estimated prevalence of GOLD stage I or higher COPD in Sousse (7.8%) (Table 4). The prevalence of self-reported doctor-diagnosed COPD was higher in females than males (4.8% (0.9) *vs.* 2.3% (0.8), respectively).

The prevalence of doctor-diagnosed COPD increased with age, particularly in men as seen in Table 4 but no clear trend was seen with increasing pack-years of smoking.

**Table 4:** Prevalence of COPD according to Doctor Diagnosis’s by; gender, age and pack years *.

	Doctor-Diagnosed COPD		
	Male	Female	Total
Age, year%			
40–49	1.1 (0.7)	4.6 (1.5)	2.8 (0.9)
50–59	2.4 (1.4)	7.0 (1.9)	4.6 (1.4)
60–69	3.2 (2.5)	3.8 (2.8)	3.5 (2.4)
70+	9.4 (6.8)	0	4.1 (3.0)
All	2.3 (0.8)	4.8 (0.9)	3.5 (0.7)
Pack-Years%			
Never-smoker	3.3 (1.5)	4.2 (0.9)	4.0 (0.8)
0–10	0	6.2 (5.7)	2.0 (2.0)
10–20	3.3 (2.5)	19.8 (17.2)	5.3 (3.4)
20+	1.8 (0.9)	9.2 (7.2)	2.3 (0.8)
Total	2.3 (0.8)	4.8 (0.9)	3.5 (0.7)

* All values are % (SE).

The prevalence of cough, sputum, wheezing, and breathlessness in patients with COPD Stages I and II are shown in Table 5. The frequency of these respiratory symptoms increased with the severity of COPD. Only 2.7% of the subjects had ever been tested by lung function tests (spirometry).

**Table 5:** Frequencies of respiratory symptoms in patients with chronic obstructive pulmonary disease.

	COPD defined as			
	LLN Stage I+ (*n* = 33)	LLN Stage II (*n* = 30)	GOLD Stage I+ (*n* = 51)	GOLD Stage II (*n* = 39)
Cough	13 (39.39)	13 (43.33)	19 (37.25)	17 (43.59)
Sputum	18 (54.55)	17 (56.67)	25 (49.02)	22 (56.41)
Wheezing	20 (60.61)	20 (60.67)	25 (49.02)	22 (56.41)
Dyspnea	12 (36.36)	12 (40.00)	16 (31.37)	16 (41.03)
Chronic cough with phlegm †	7 (21.21)	7 (23.33)	10 (19.61)	9 (23.08)

Values are n (%). Definition of abbreviations: COPD = chronic obstructive pulmonary disease; GOLD = Global Initiative for Chronic Obstructive Lung Disease; LLN = Lower Limit of Normal; † Cough with phlegm for at least 3 months per year in the previous 2 years.

## 4. Discussion

The key findings of this population-based prevalence survey are that 7.8% of the residents of Sousse, Tunisia, 40 years of age or over had at least Stage I COPD, and this was more common in men than in women. These findings indicated COPD as a more serious public health problem in Tunisia than expected from previous studies [3] and illustrate the magnitude of the burden that COPD will pose in the near future, as the proportion of the population living into old age when chronic diseases including COPD are common.

Our finding is consistent with an expected range of 4% to 10% from an international review of COPD prevalence based on spirometry [8,18,19]. As expected, COPD prevalence found in our study most likely reflects the aging of our study population. This is similar to the results found in many other countries using the same BOLD methodology [14] and in many other previous epidemiological studies [19,20]. Indeed, the projected increase in the prevalence of COPD worldwide is being driven more by the projected aging of the world population than by estimated changes in the prevalence of smoking [21]. Demonstrating this point, our data show a steep gradient in COPD prevalence with increasing age, with the highest prevalence seen in men and women ≥70 years of age. This result reflects the use of the threshold based on a fixed ratio of less than 0.70 to define irreversible airflow obstruction as recommended by the GOLD. Indeed, the fixed ratio has been shown to overdiagnose airflow obstruction, especially in the elderly since it has a small but significant age-related regression [17,22,23].

The finding that COPD prevalence increased with age does not minimize the fact that smoking is an important risk factor for COPD [4,24,25]. In the present study, smoking 10+ pack years per year, was independently associated with an increased risk of COPD. 

However it is surprising that in the present study, half of patients with COPD (50%) were never smokers. The prevalence of COPD in never smokers, which was as high as 3.9%, was much higher in comparison to other countries participating in the BOLD Study [14] and suggested that factors other than smoking exposure might also be involved in COPD.

Moreover, the potential risk factors we explored were not associated with having more COPD (*i.e.*, exposure to occupational dust) suggesting that other factors that were not explored on our model should be considered. There is a lot of debate in the current literature whether exposure to biomass cooking may be a risk factor for COPD, particularly in low income settings, however the evidence is contradictory [26]. 

As reported in the study of Lamprecht *et al.*, we found a consistent association of airflow obstruction in never smokers with asthma and older age [27]. Similar results were found in two other cross-sectional studies that showed that COPD in never smokers was more common in older subjects with a medical diagnosis of asthma and with a low educational level [27,28]. Other studies [29,30] are consistent with our finding and have found that persons who could have or have had an asthma will be progressed into chronic obstruction. Thus, asthma has been identified as a risk factor of COPD [30].

In Tunisia, the COPD prevalence in women is lower than that seen in men. This situation is probably due to the fact that Tunisian women have not been as likely to smoke as men. This situation is different in some developed countries, where the prevalence of smoking in women is now often as high as that in men [19]. There has been considerable controversy as to whether women are at equal or perhaps at greater risk than men given an equal exposure. This controversy has not been resolved, although there is increasing evidence that women may be more vulnerable [21]. In developing countries, the increase in smoking among women, that is likely to occur, will probably lead to a tidal wave of COPD as women both have more exposure and live longer. Women are also more likely than men to be exposed to high indoor air pollution levels in developing countries. Thus, fossil fuel pollution has been found to have a greater effect in women compared with men [31,32]. 

Surprisingly, we found that a low BMI is associated with having more COPD, and why this should be the case and whether other related factors such as nutrition could explain this finding, warrants further investigation.

Our results revealed only 3.5% of participants which reported doctor-diagnosed COPD. An important finding of our study is that there was a huge gap between physician diagnosis of COPD and the presence of airflow obstruction defined by spirometry. Moreover, more than 87.9% had never been diagnosed before this survey. This suggests that diagnosis of COPD based on symptoms may not be adequate and awareness of COPD among health professionals require more use of objective measures of lung function to confirm the diagnosis.

Prevalence estimates depend on the diagnostic criteria and methods used [33]. In order to obtain accurate estimates of COPD prevalence, we used standardized methods developed by the BOLD initiative [13] that incorporate many quality control measures, including careful population-based sampling with high response rate, standardized spirometry equipment, central training, certification, and monitoring of technicians, over reading of all spirograms and a strict protocol for the translation of questionnaires.

Estimated population frequency of COPD in Stage ≥1 in our study was very high in subjects aged 70 or more (about 61.5% in men and over 12.5% in women). Using a post-bronchodilator fixed FEV1/FVC ratio of less than 0.7 as a threshold for COPD diagnosis in this age group can probably lead to overestimation of the disease prevalence. Indeed, the limitations of using a fixed FEV1/FVC ratio <0.70 as a cut point for airflow obstruction, as recommended by the GOLD, have been highlighted recently [22,34,35] and this use has the potential to misclassify at older ages, since the ratio has a small but significant age-related regression [22]. The present controversy revolves around the question of whether using a fixed ratio of FEV1/FVC or a more statistically appropriate metric, such as the lower limit (e.g., 95th percentile) of the population distribution is a better way to separate normal aging from abnormal aging (*i.e.*, disease). A population-based study in individuals over 70 years showed FEV1/FVC ratio below 0.7 in about 35% of asymptomatic, non-smoking subjects [6]. Study based on the NHANES III data has shown that up to 20% of elderly subjects with FEV1/FVC above 5th percentile had FEV1/FVC ratio below 0.7 [35].

The LLN, based on the normal distribution, classify the bottom 5% of the healthy population as abnormal. When we use LLN criterion in the evaluation of FEV1/FVC, it could be one of alternatives to minimize the potential misclassification [15,36]. Several previous studies showed that use of the LLN criterion instead of the fixed ratio criterion minimizes known age biases and better reflects clinically significant irreversible airway obstruction [22,35].

Compared to the reports on COPD prevalence using the same methods [8,37,38], our data showed a lower prevalence of COPD in Tunisia than that in the five Latin American cities [8], in South Africa [37] and in Turkey [38]; indeed, GOLD Stage II COPD, constituted about half of all the COPD cases (4.2% overall; 7.0% in men and 1.2% in women).

The high prevalence of GOLD Stage II COPD in our population could probably be attributed to the fact that all the measured FEV1 and FVC values were expressed relative to the NHANES III white American references values; however, the Tunisian spirometry reference values are 10% lower than Caucasians and the latter may result in an over-diagnosis of GOLD Stage II COPD [39].

## 5. Conclusions

The results of BOLD Study carried out in Tunisia confirm the high prevalence of COPD and call for more research to be directed toward preventive measures and efforts. In fact, smoking cessation and early diagnosis may inhibit the growth to a relevant clinical stage. Therefore, health-care professionals are duty to do more researches, to inform patients about the disease and to advise them to reduce and even halt smoking. Hence, the outbreak of COPD may be monitored.
